# A Comparison Between Two-Dimensional and Three-Dimensional Endoscopic Imaging for Breast Augmentation

**DOI:** 10.7759/cureus.77889

**Published:** 2025-01-23

**Authors:** Ben Li Chan, Jui Hsing Wu, Lee Won

**Affiliations:** 1 Surgery, Style Aesthetic Clinic, Taichung, TWN; 2 Surgery, For Sure Plastic Surgery Clinic, Taipei, TWN; 3 Surgery, E1 Plastic Surgery Clinic, Anyang, KOR

**Keywords:** 2d imaging, 3d imaging, 3d imaging endoscopic surgery, breast augmentation, reoperation

## Abstract

Background

This study aimed to compare the reoperation rates between two-dimensional (2D) and three-dimensional (3D) endoscopic imaging used for breast augmentation.

Methodology

The medical records of females who underwent primary or revision breast augmentation surgery at our clinic from January 2014 to December 2023 were retrospectively reviewed. From January 2016 to August 2022, endoscopic operations were performed under 2D imaging, and from September 2022 onward, operations were performed using a novel synthetic 3D imaging technique (Monostereo®). All operations were performed by the same surgeon. The reoperation rate and other study variables between the two groups were compared.

Results

A total of 351 female patients (median age = 33 years) were included, of whom 216 had surgery using 2D imaging and 135 had surgery using 3D imaging. The overall reoperation rate was 4.3% (15/351), and it was significantly lower in the 3D imaging group compared to the 2D imaging group (14/216 (6.5%) vs. 1/135 (0.7%); p = 0.01). Age, incision site, placement, implant surface type, or surgery time had no association with reoperation. However, patients who required reoperation had a significantly larger implant size (330 (300-360) mL) than those who did not require a reoperation (275 (270-325) mL) (p < 0.05). Capsular contracture was the reason for 60% (n = 9) of the reoperations, and 40% (n = 6) of the reoperations were a size change requested by the patients.

Conclusions

Breast augmentation using the novel synthetic 3D imaging technique is associated with a lower reoperation rate compared to conventional 2D imaging. Further investigation is needed to examine the benefits of 3D imaging for breast augmentation.

## Introduction

Breast augmentation is a commonly performed aesthetic operation, and, in most cases, an endoscopic approach is used [1]. Reoperation following primary augmentation is associated with 2.8% to 20.4% capsular contracture rates depending on various factors and study methodologies [1,2]. The two most common reasons for reoperation are capsular contraction and implant malposition [1,3]. Several studies have examined risk factors for capsular contraction, including the plane the implant is placed in, the texture of the implant, antibiotic irrigation, and the development of a hematoma/seroma [4-10]. While most of these factors are not related to surgical technique, improvements in surgical methods may help reduce the risk of postoperative contracture.

The development of endoscopic surgical methods has revolutionized surgery, and minimally invasive endoscopic procedures are used in every surgical discipline. Endoscopic surgery offers the advantages of less surgical trauma, faster recovery, less postoperative pain, and improved cosmesis compared to open surgical procedures. However, a major challenge of endoscopic surgery is that the surgeon must manipulate instruments and tissues within a three-dimensional (3D) environment while relying on a two-dimensional (2D) monitor for visualization. Additionally, there is a relatively steep learning curve for developing the hand-eye coordination required for these procedures. Even after achieving proficiency, maintaining depth perception and accurate visualization remains highly demanding for the surgeon [11]. This challenge can lead to intraoperative errors, even among highly skilled surgeons [11].

As technology has progressed, in the past decade, various methods of 3D laparoscopic imaging have been developed [11-14]. Autostereoscopic 3D imaging offers an improved sense of depth and superior image quality compared to conventional 2D imaging [12,13], thereby enhancing the precision and outcomes of endoscopic and laparoscopic surgeries [12,15,16]. Although this technology is relatively new, it has already been linked to better outcomes than traditional 2D endoscopy across various surgical specialties.

Earlier 3D imaging systems often relied on dual-camera setups or microscopic arrays of lenses to generate stereoscopic effects [17], which presented challenges such as the potential for additional incisions and a steeper learning curve for surgeons. In contrast, the synthetic 3D imaging system (Monostereo® used in this study) represents a significant advancement by addressing these limitations through an innovative 2D to 3D conversion technology. This system seamlessly transforms standard 2D signals into stereoscopic images without requiring specialized hardware, such as dual cameras or lens arrays. Accordingly, it minimizes the need for additional training and steep learning curves, ensuring that surgery duration and workflow efficiency remain unaffected [18]. The present study aimed to compare the reoperation rates of breast augmentation surgeries performed using the synthetic 3D system versus conventional 2D imaging.

## Materials and methods

The records analyzed in this study were retrospective, de-identified data that did not include any personally identifiable information. All data were handled with strict confidentiality and in compliance with applicable healthcare privacy regulations during analysis.

Patients

We retrospectively reviewed the medical records of female patients who underwent primary or revision breast augmentation surgery at our clinic from January 2014 to December 2023. Inclusion criteria were age ≥20 years and a silicone breast implant. Patients diagnosed with malignancy or with missing data were excluded. Age and surgical data including incision site, implant size, and surgical time were collected. All patients were followed up for at least one year, and data of patients who required a reoperation were compared with those who did not require a reoperation.

From January 2016 to August 2022, endoscopic operations were performed under 2D imaging, and from September 2022 onward, operations were performed under 3D imaging. A 2D to 3D video converter (Monostereo®, MedicalTek, Taiwan) was connected to a standard 2D endoscope to provide synthetic 3D images. All surgeries were performed by the same surgeon (BLJ).

Synthetic three-dimensional imaging using Monostereo® technology

**Figure 1:**
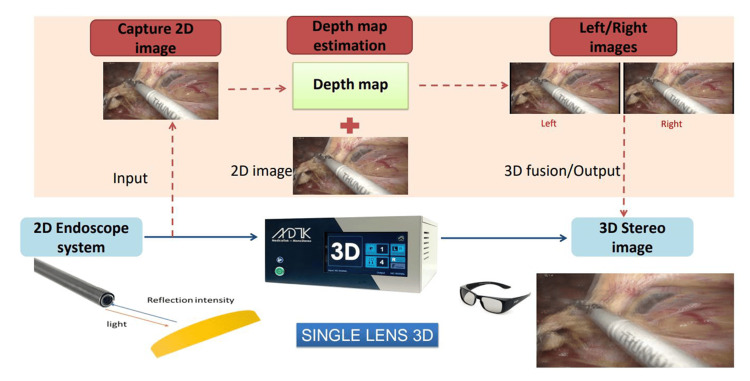
Schematic illustration of the operating mechanism of the Monostereo® 3D system utilizing an innovative 2D to 3D conversion technology.

The synthetic 3D imaging technique used in this study employed a novel 2D to 3D video converter (Monostereo®, MedicalTek, Taiwan). This system connects to a standard 2D endoscope and enables real-time conversion of 2D video into stereoscopic 3D imaging. It features on-demand switching between 2D and 3D modes with a single click and adjustable disparity levels to customize the depth effect. Additionally, the system has been shown to reduce common 3D-induced symptoms of the surgeons, such as dizziness and headache [18]. Without the need for specialized multiple lenses, this technology provides a cost-effective solution that enhances depth perception and eliminates the need for additional training, significantly reducing the learning curve compared to earlier 3D systems. The schematic illustration of the mechanism of Monostereo® is shown in Figure 1.

Endoscopic breast augmentation procedure

Consistent with traditional breast augmentation, the breast augmentation begins with a 3 cm incision in the axillary fold, following anesthetic injection with 2% lidocaine and epinephrine to prepare the axillary incision site. The pectoralis major muscle is then identified, and its insertion serves as a guide for creating space to accommodate the endoscope. Once the surgical plane between the pectoralis major and minor muscles is established, approximately 200 cc of tumescent fluid is injected into each side. 2D/3D endoscopic imaging allows for precise identification of the plane between the pectoralis major and minor muscles, revealing distinct areolar tissue within a non-bleeding space, thereby reducing the risk of intraoperative hemorrhage.

**Figure 2:**
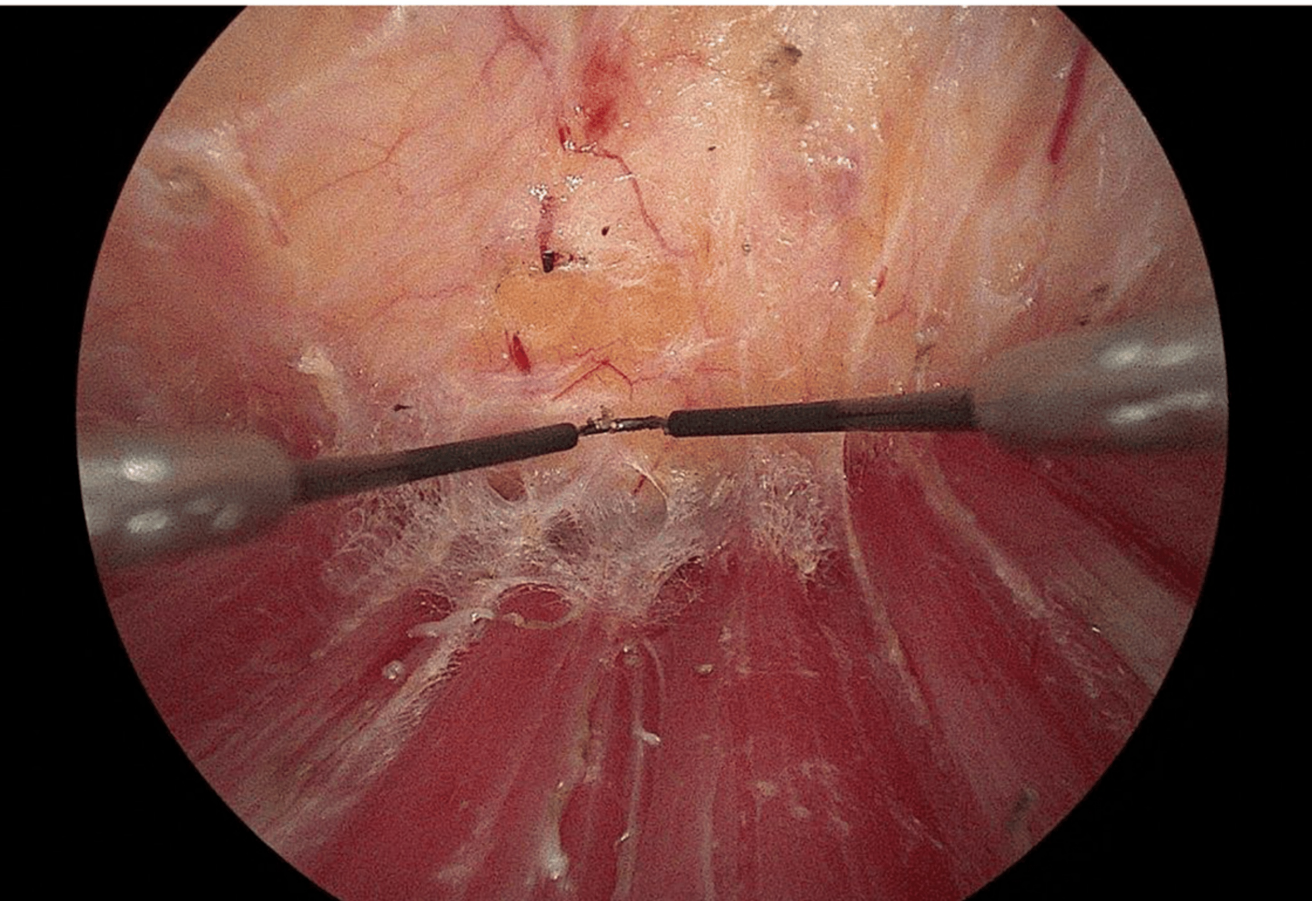
Two-plane technique involving dissecting the space between the pectoralis major and minor muscles.

The procedure employs a two-plane technique, which involves dissecting the space between the pectoralis major and minor muscles (Figure 2). Dissection starts in the inferior-medial region until the pectoralis major muscle insertion is visible, allowing for resection to facilitate dissection augmentation. The lateral border of the pectoralis major is then identified, separated, and resected from lateral to medial. If performed within an avascular space, minimal bleeding is observed. In some cases, part of the lateral muscle is left intact to prevent outward implant displacement, while complete severance of the muscle is done if a fuller chest is desired by the patient.

Following the dissection of the pectoralis major muscle, the lateral tissue is carefully separated. To prevent the development of a wide cleavage, only partial dissection of the pectoralis major is performed on the medial side, leaving some muscle intact. This completes the unilateral space dissection. After the two-plane dissection, the fascia beneath the breast becomes clearly visible. Based on the patient’s needs and the desired surgical outcome, the fascia is adjusted to the appropriate position to create the intended two-plane dissection space. Any bleeding vessels encountered are promptly cauterized, as observed via 2D/3D endoscopic imaging. To prevent future implant displacement, a midline incision is made in the pectoralis major muscle. The wound is irrigated with Adam’s solution, the implant is inserted, and the process is repeated on the opposite side.

Statistical analysis

Continuous data without a normal distribution were presented as the median (interquartile range (IQR)) and were analyzed by the Wilcoxon rank-sum test. Normally distributed continuous data were compared with the Shapiro-Wilk test. Categorical data were analyzed by the chi-square test or Fisher’s exact test, as appropriate, and presented as count (%). A two-sided p-value <0.05 was regarded as statistically significant. Data management and statistical analyses were conducted by using SAS version 9.4 software (SAS Institute, Inc., Cary, NC, USA).

## Results

Patient characteristics

A total of 351 female patients were included in the final analysis, of whom 216 had surgery performed using 2D imaging and 135 had surgery performed using 3D imaging. Surgical and implant characteristics are presented in Table 1. The median patient age was 33 years. Compared to the 2D imaging group, the 3D imaging group had significantly higher frequencies of transaxillary incision (79/135 (58.5%) vs. 78/216 (36.1%); p < 0.001), subfascial placement (42/135 (31.1%) vs. 34/216 (15.7%); p = 0.001), a smooth silicone implant (102/135 (76.1%) vs. 86/216 (40.2%); p < 0.001), and a larger implant size (300 mL (275-335 mL) vs. 350 mL (330-375 mL); p < 0.001). There were no postoperative infections or hematomas in either group.

**Table 1:** Patient and surgical characteristics.

Variable	Total (N = 351)	2D imaging (n = 216)	3D imaging (n = 135)	P-value
Age (years)	33.0 (29.0–39.0)	33.0 (29.0–39.0)	34.0 (29.0–39.0)	0.814
Incision site				
Periareolar	183 (52.1)	129 (59.7)	54 (40.0)	<0.001
Inframammary	6 (1.7)	4 (1.9)	2 (1.5)	
Transaxillary	157 (44.7)	78 (36.1)	79 (58.5)	
Transumbilical	5 (1.4)	5 (2.3)	0 (0.0)	
Placement				
Subfascial	76 (21.7)	34 (15.7)	42 (31.1)	0.001
Two-plane	275 (78.3)	182 (84.3)	93 (68.9)	
Implant surface texture				
Smooth	188 (54.0)	86 (40.2)	102 (76.1)	<0.001
Textured	160 (46.0)	128 (59.8)	32 (23.9)	
Data missing	3	2	1	
Implant size (mL)	330.0 (300.0–360.0)	300.0 (275.0–335.0)	350.0 (330.0–375.0)	<0.001
Outcome				
Surgery time (hours)	3.5 (3.0–4.0)	3.5 (3.0–4.0)	3.5 (3.0–4.0)	0.098
Reoperation	15 (4.3)	14 (6.5)	1 (0.7)	0.01
Time (months)	31 ± 22	32 ± 23	20	-

The overall reoperation rate was 4.3% (15/351), and the reoperation rate was significantly lower in the 3D imaging group compared to the 2D imaging group (1/135 (0.7%) vs. 14/216 (6.5%); p = 0.01).

Associations between reoperation and study variables are shown in Table 2. Of the 15 patients who required reoperation, one was in the 3D group and 14 were in the 2D group (p = 0.010). Age, incision site, placement, implant surface type, or surgery time had no association with reoperation. However, patients who required reoperation had a significantly larger implant size (330 mL (300-360 mL)) than those who did not require a reoperation (275 mL (270-325 mL)) (p < 0.05). Capsular contracture was the reason for 60% (n = 9) of the reoperations, and 40% (n = 6) of the reoperations were a size change requested by the patients.

**Table 2:** Associations between reoperation and study variables.

Study variables	Total	Reoperation		P-value
		Yes (n = 15)	No (n = 336)	
Imaging				
2D	216 (61.5)	14 (93.3)	202 (60.1)	0.01
3D	135 (38.5)	1 (6.7)	134 (39.9)	
Age (years)	33 (29-39)	32 (29-40)	33 (29-39)	0.837
Incision site				
Periareolar	183 (52.1)	8 (53.3)	175 (52.1)	1
Inframammary	6 (1.7)	0 (0.0)	6 (1.8)	
Transaxillary	157 (44.7)	7 (46.7)	150 (44.6)	
Transumbilical	5 (1.4)	0 (0.0)	5 (1.5)	
Placement				
Subfascial	76 (21.7)	5 (33.3)	71 (21.1)	0.332
Dural plane	275 (78.3)	10 (66.7)	265 (78.9)	
Implant surface				
Smooth	188 (54.0)	8 (53.3)	180 (54.1)	0.956
Textured	160 (46.0)	7 (46.7)	153 (45.9)	
Implant size (mL)	330.0 (300–360)	330.0 (300–360)	275 (270–325)	0.004
Outcome				
Surgery time (hours)	3.5 (3–4)	4 (3–5)	3.5 (3–4)	0.076

## Discussion

The primary result of this study is that using the novel synthetic 3D endoscopic imaging system is associated with a significantly lower reoperation rate in patients undergoing breast augmentation compared to conventional 2D imaging. Of the variables examined, only larger implant size was correlated with reoperation. Notably, the primary reason for reoperation was capsular contraction (60%), followed by a patient requesting an implant size change.

All of the procedures in this report were performed by the same surgeon, with 2D imaging used during the earlier period, and 3D imaging used during the later period. The use of 3D endoscopic imaging provides an improved sense of stereoscopic depth and image quality compared to conventional 2D imaging and thus allows manipulation of instruments and tissues to be more like the experience of open procedures [11], and the results of this study suggest that this better visualization is associated with a lower reoperation rate.

Since the development and introduction of 3D laparoscopic/endoscopic imaging, its value has been examined in many different surgical fields, and overall the results have been positive. In a recent report, Yang et al. [14] compared the outcomes of 3D and 2D laparoscopy for radical hysterectomy and pelvic lymphadenectomy for treating early-stage cervical cancer. Using the 3D system significantly shortened operation time and blood loss. In addition, the surgeons commented that the 3D system improved depth perception and precision, and reduced eye strain of the operators. Recent studies have also examined 3D imaging in the treatment of breast cancer. Lai et al. [19] reviewed the records of 70 patients who received 80 single-port 3D videoscope-assisted endoscopic nipple-sparing mastectomies for breast cancer. The overall results showed the procedure was safe and efficient and was associated with good aesthetic outcomes. About 90% of the patients were very satisfied with the aesthetic results, and said they would choose the same procedure in the future if it was required. Notably, the analysis indicated that about 14 cases were needed for a surgeon to become familiar with the technique. A similar study of 145 breast cancer and gynecomastia cases also reported high patient satisfaction and a conclusion by the authors that the procedure can be performed safely in either malignant or benign breast conditions and offers promising cosmetic results [20].

Several meta-analyses have been done examining 3D imaging for different types of surgeries. Huang et al. [19] compared the results of 3D and 2D endoscopic thyroidectomy that included 15 studies and 1,190 patients and concluded that 3D endoscopic thyroidectomy is an efficient, safe, and reliable method with better depth perception and stereoscopic vision, and outcomes similar to those using 2D imaging. Similarly, a systematic review and meta-analysis of 3D laparoscopy for the treatment of colorectal cancer concluded that 3D laparoscopy was associated with less blood loss, shorter time to postoperative flatus, shorter operation time, and shorter postoperative hospital stay, as well as an increased number of dissected lymph nodes [21]. Similar meta-analyses have shown that 3D imaging provides benefits in endonasal surgery [22], training novice surgeons [23], and better results compared to ultra-high-definition laparoscopic technology [24].

It should be noted that in this study reoperation was correlated with larger implant size. However, the correlation between implant size and reoperation is inconclusive, warranting further investigation. Calobrace et al. [5] reported that implant size <355 mL was a significant risk factor for capsular contracture, but Henriksen et al. [6] reported that implant size >350 mL increased the risk of complications requiring surgical intervention. As this study was primarily focused on the use of 3D visualization it is difficult to draw any conclusions regarding these results.

Limitations

The study has several limitations, primarily due to its retrospective design. Second, the small sample size may cause a bias in data generalization and interpretation. Third, the follow-up duration may not be sufficient to accurately capture the reoperation rate. A further study with a larger cohort and longer follow-up is needed to clarify the benefit of 3D image operation.

## Conclusions

Breast augmentation using the synthetic 3D imaging system is associated with a lower reoperation rate compared to conventional 2D imaging. Further investigation is needed to examine the benefits of 3D imaging for breast augmentation.
